# Longitudinal tau and metabolic PET imaging in relation to novel CSF tau measures in Alzheimer’s disease

**DOI:** 10.1007/s00259-018-4242-6

**Published:** 2019-01-04

**Authors:** Antoine Leuzy, Claudia Cicognola, Konstantinos Chiotis, Laure Saint-Aubert, Laetitia Lemoine, Niels Andreasen, Henrik Zetterberg, Keqiang Ye, Kaj Blennow, Kina Höglund, Agneta Nordberg

**Affiliations:** 1 Department of Neurobiology, Care Sciences and Society, Division of Clinical Geriatrics, Karolinska Institutet, Stockholm, Sweden; 2 Department of Psychiatry and Neurochemistry, Institute of Neuroscience and Physiology, The Sahlgrenska Academy, University of Gothenburg, Mölndal, Sweden; 3 ToNIC, Toulouse NeuroImaging Center, University of Toulouse, Inserm, UPS, Toulouse, France; 4 Nuclear Medicine Department, University Hospital of Toulouse, Toulouse, France; 5 Theme Aging, Karolinska University Hospital, Stockholm, Sweden; 6 Clinical Neurochemistry Laboratory, Sahlgrenska University Hospital, Mölndal, Sweden; 7 Department of Neurodegenerative Disease, UCL Institute of Neurology, Queen Square, London, UK; 8 UK Dementia Research Institute at UCL, London, UK; 9 Pathology & Laboratory Medicine, Experimental Pathology, Emory University School of Medicine, Atlanta, GA USA

**Keywords:** Alzheimer’s disease, Tau, CSF, PET imaging, [^18^F]FDG, [^18^F]THK5317

## Abstract

**Purpose:**

Studies comparing CSF and PET tau biomarkers have included only commercial CSF assays examining specific phosphorylation sites (e.g. threonine 181, P-tau_181p_) and mid-domain tau (i.e. total tau, T-tau). Moreover, these studies did not examine CSF tau levels in relation to cerebral glucose metabolism. We thus aimed to examine CSF tau measures, using both commercial and novel assays, in relation to [^18^F]THK5317 (tau) and [^18^F]FDG PET (glucose metabolism).

**Methods:**

Fourteen Alzheimer’s disease (AD) patients (seven prodromal, seven dementia) underwent [^18^F]THK5317 and [^18^F]FDG PET studies, with follow-up performed in ten subjects (six prodromal, four dementia) after 17 months. In addition to commercial assays, novel measures capturing N-terminus+mid-domain (tau N-Mid) and C-terminally truncated (tau-368) fragments were included.

**Results:**

While the levels of all forms of CSF tau were found to be inversely associated with baseline [^18^F]FDG uptake, associations with baseline [^18^F]THK5317 uptake varied in relation to the degree of isocortical hypometabolism ([^18^F]FDG SUVR). Changes in the levels of the novel CSF markers tracked longitudinal changes in tracer uptake better than changes in P-tau_181p_ and T-tau levels, and improved concordance with dichotomized regional [^18^F]THK5317 measures.

**Conclusion:**

Our findings suggest that neurodegeneration may modulate the relationship between CSF and PET tau biomarkers, and that, by comparison to P-tau_181p_ and T-tau, tau-368 and tau N-Mid may better capture tau pathology and synaptic impairment.

**Electronic supplementary material:**

The online version of this article (10.1007/s00259-018-4242-6) contains supplementary material, which is available to authorized users.

## Introduction

Although many details surrounding the pathogenesis of Alzheimer’s disease (AD) remain unclear, a dominant viewpoint is that the dysmetabolism of amyloid-β (Aβ) initiates tau pathology that results in neurodegeneration and cognitive decline. Cerebrospinal fluid (CSF) measures of tau include phosphorylated tau (P-tau), an indicator of an abnormal state associated with the formation of neurofibrillary tangle (NFT) pathology, and total tau (T-tau), that reflects the intensity of axonal and neuronal damage [1]. Recently, positron emission tomography (PET) ligands selective for AD-related paired helical filament (PHF) tau present in NFTs have been introduced into the field.

One such ligand is [^18^F]flortaucipir (formerly known as [^18^F]T-807 and [^18^F]AV1451) [2, 3], and studies examining the relationship between [^18^F]flortaucipir uptake and CSF tau levels have shown modest positive correlations in cognitively unimpaired older adults and in patients with AD and non-AD neurodegenerative disorders [4–7]. These studies, however, used commercial assays that use monoclonal antibodies directed against specific phosphorylation sites (e.g. threonine-181, P-tau_181p_) and the mid-region of tau (i.e. T-tau) [8, 9]. Increasing evidence, however, indicates the presence of tau fragments spanning both the mid-domain and various terminal regions [10], suggesting that assays targeting specific variants of tau may be required to more fully characterize the relationship between CSF and PET tau biomarkers. Moreover, these studies did not examine the link between CSF tau levels and [^18^F]FDG PET, an important measure given evidence indicating that synaptic integrity mediates the impact of tau on cognition [11].

The aims of this study were thus to investigate the association between CSF tau levels, derived from both commercial assays (P-tau_181p_ and T-tau) [8, 9] and novel assays, the latter including N-terminus plus mid-domain regions (tau N-Mid) and C-terminally truncated tau (tau-368), and longitudinal findings using the tau PET tracer [^18^F]THK5317 [(*S*)-[^18^F]THK5117] [12–14] and [^18^F]FDG in a sample of patients with AD. Given findings suggesting that disease progression may be accompanied by a reduction in CSF tau [15, 16], it was hypothesized that the degree of glucose hypometabolism may modulate associations between CSF tau levels and [^18^F]THK5317 uptake. Further, in light of the likely future clinical use of tau imaging, we also aimed to examine the agreement between dichotomized CSF tau levels and [^18^F]THK5317 uptake.

## Materials and methods

### Study sample

Fourteen patients with AD (seven prodromal AD, seven AD dementia), and nine cognitively unimpaired individuals (THK-controls; five young, 20–30 years old, four elderly, 58–71 years old) who had previously participated in baseline investigations were included [11, 17]. Follow-up [^18^F]THK5317 and [^18^F]FDG PET data were acquired in ten AD patients (six prodromal, four dementia) after a median of 17 months (interquartile range, IQR, 15–18 months) [18]. Baseline cross-sectional [^18^F]THK5317 and [^18^F]FDG PET studies were performed within 1.5 months (IQR 1–2.7 months) and follow-up studies within 0.5 months (IQR 0–1 months). CSF samples were collected approximately 24 months before the baseline [^18^F]THK5317 PET studies (IQR 14–34.5 months). Owing to outlying CSF tau values, one patient with AD dementia was only included in regression analyses involving CSF ratio measurements.

All patients had originally undergone clinical assessment at Theme Aging, Karolinska University Hospital, Stockholm, Sweden, and underwent follow-up, as detailed elsewhere [17]. Patients diagnosed with AD dementia fulfilled the criteria for probable AD and showed in vivo evidence of Aβ pathology with an abnormal [^11^C]Pittsburgh compound B [^11^C]PIB) scan [19]. Patients diagnosed with prodromal AD met the criteria for mild cognitive impairment [20] and showed an abnormal [^11^C]PIB scan [21]. THK-controls were recruited through Clinical Trial Consultants AB (Uppsala University Hospital, Uppsala, Sweden) or from among patients’ relatives; all were nonsmokers and not taking medication, completed extensive clinical evaluations, and at inclusion had no cognitive complaints, prior head injury or known neurological/psychiatric disorders. Elderly THK-controls were [^11^C]PIB-negative, and showed no differences in regional [^18^F]THK5317 binding as compared with young THK-controls.

Informed consent was obtained from all individual participants included in the study. Ethical approval was obtained from the Regional Human Ethics Committee of Stockholm and the Faculty of Medicine and Radiation Hazard Ethics Committee of Uppsala University Hospital, Uppsala, Sweden. A further 15 individuals were included as CSF biomarker-negative controls (CSF-controls); these were patients with minor cognitive complaints but with basic (cell count, albumin ratio, IgG index) and core (Aβ_1-42_/Aβ_1-40_, P-tau_181p_, and T-tau) CSF biomarkers within normal ranges. The CSF samples used were deidentified leftover aliquots from routine clinical analyses, following a procedure approved by the Ethics Committee of the University of Gothenburg (EPN 140811).

### CSF measurements

CSF tau was quantified in the Clinical Neurochemistry Laboratory of the University of Gothenburg, Mölndal, Sweden, using three approaches: commercially available INNOTEST enzyme-linked immunosorbent assay (ELISA) kits (Fujirebio) for P-tau_181P_ and T-tau, an in-house ELISA targeting tau species from the N-terminus to the mid-domain of tau (tau N-Mid), and a single molecule array (Simoa) method targeting a C-terminally truncated form of tau ending at amino acid 368 (tau-368) [22]. Aβ_1-42_ and Aβ_1-40_ were determined using the Meso Scale Discovery Abeta Triplex immunoassay (MSD, Gaithersburg, MD) according to the kit insert. Additional methodological details, including those pertaining to the validation of the tau N-Mid and tau-368 assays, are included in Online Resource 1.

### PET data acquisition and analysis

Dynamic [^18^F]THK5317 PET studies were performed over 60 min following intravenous bolus injection of 217 ± 42 MBq. A static 15 min [^18^F]FDG scan was performed 30 min after injection of 3 MBq/kg. Baseline and follow-up images were coregistered to their respective T1-weighted MR images using PMOD v. 3.5 (PMOD Technologies., Zurich, Switzerland). In order to account for possible spillover effects from the white matter, MRI-based partial volume correction was applied to [^18^F]THK5317 PET images, as implemented in PMOD [23].

Distribution volume ratio (DVR) images were calculated for [^18^F]THK5317 using the cerebellar cortex as the reference tissue [12, 24]. For [^18^F]FDG [18], standardized uptake value ratio (SUVR) was used as the outcome measure using the pons as the reference region.

Following segmentation of T1 images using SPM8, the inverse transformation parameters generated were used to spatially warp a probabilistic atlas into native space in order to sample the parametric images [25]. Bilateral grey matter regions of interest (ROIs) were based on previously published findings with [^18^F]THK5317 [17], and included the frontal, temporal (medial, lateral aspects), parietal, posterior cingulate and occipital cortices, as well as an isocortical composite ROI comprising these regions minus medial temporal structures. Two additional composite regions approximating the Braak staging scheme for tau pathology [26] were also included: a limbic ROI (Braak III/IV) comprising the hippocampus, parahippocampus, amygdala, fusiform and middle inferior temporal gyri, orbital and straight frontal gyri, parietal-temporal-occipital junctions and the temporal poles, and an isocortical ROI (Braak V/VI) comprising all isocortical regions except the precentral and postcentral gyri. Annual rates of change in [^18^F]THK5317 DVR and [^18^F]FDG SUVR were calculated as follows: (follow-up value − baseline value)/between-scan interval (years) [18].

### Determination of regional [^18^F]THK5317 and CSF tau cut-off values

CSF P-tau_181p_ and T-tau values were classified as abnormal using cut-off values of >80 pg/mL and > 400 pg/mL, respectively [27]. Unpublished internal cut-off values of >272 pg/mL and <0.038 were used for tau N-Mid and tau-368/T-tau, respectively. These were determined using receiver operating characteristic (ROC) analysis (highest combined sensitivity and specificity) among 50 biomarker-positive AD patients (low Aβ_1-42_, high P-tau_181p_ and T-tau) and 50 cognitively unimpaired biomarker-negative individuals. Regional [^18^F]THK5317 DVR cut-off values were defined using the mean plus two standard deviations of the DVR values in the THK-control group (elderly and young combined), in agreement with previous approaches for both tau [6] and Aβ PET [28]. Given the lack of cut-off values for tau-368 and tau-368/tau N-Mid, as well as the absence of controls with both CSF and [^18^F]THK5317 data, concordance using these measures was not investigated.

### Statistical analysis

Patient characteristics and CSF measures were compared using the Mann-Whitney *U* test. To examine the relationship between regional PET ([^18^F]THK5317 DVR and [^18^F]FDG SUVR) and CSF measures, nonparametric rank-based estimation multilinear regression models were implemented [29]. Here, PET measures were regressed onto CSF values, adjusting for age, ventricular volume and the time between the CSF and PET investigations. For models examining CSF tau values in relation to baseline [^18^F]THK5317 DVR, isocortical composite [^18^F]FDG SUVR, included as an interaction term with CSF tau, was binarized using a median value of 1.4. Simple slope analyses were used to assess multiple linear regression two-way interactions [30]. All analyses were performed using R, v.3.3.2, with significance set at *p* < 0.05, two-tailed. ROC-derived areas under the curve (AUC) for CSF tau measures were compared between AD patients and CSF-controls using the pROC package, v.1.10 [31].

## Results

Demographic, clinical and CSF data are presented in Table 1. As expected, baseline Mini-Mental State Examination (MMSE) scores were significantly lower in AD dementia patients than in prodromal AD patients (*p* < 0.01). MMSE scores at follow-up were significantly lower than scores at baseline, in prodromal AD patients (*p* < 0.01), and across prodromal AD and AD dementia patients combined (*p* < 0.01). Tau-368/T-tau ratios were lower in AD dementia patients than in prodromal AD patients (*p* < 0.05).

**Table 1:** Demographic, clinical and CSF data

	Prodromal AD	AD dementia	AD combined
Number of patients			
Baseline	7	7	14
Follow-up	6^a^	4	10
Age at baseline (years)	65 [62, 73.5]	64 [59, 66]	65 [59.3, 74]
Male, *n* (%)	2 (29)	2 (29)	4 (29)
APOE ε4, *n* (%)	4 (57)	5 (71)	9 (64)
MMSE score			
Baseline	29 [28, 29.5]	23 [23, 24.5]^b^	25.5 [23.3, 28.8]
Follow-up, median [IQR]	25 [24, 27]^c^	20 [17, 23.8]	24 [18.5, 25]^c^
[^11^C]PIB (composite SUVR)	1.72 [1.68, 1.87]	1.74 [1.68, 1.88]	1.73 [1.67, 1.88]
Aβ_1–42_ (pg/mL)	236.1 [217, 453]	202.2 [162.4, 310.3]	218 [193, 379]
P-tau_181p_ (pg/mL)	68 [47, 76]	58 [54, 98]	63 [47, 87]
T-tau (pg/mL)	538 [346, 683]	529.1 [455, 815]	534 [372, 755]
Tau N-Mid (pg/mL)	213 [118, 294]	275 [135, 420]	240 [135, 355]
Tau-368 (pg/mL)	12.3 [11, 14.2]	9.57 [8.4, 14.8]	12.1 [9.4, 14.5]
Tau-368/T-tau	0.026 [0.02, 0.032]	0.019 [0.018,0.022]^b^	0.018 [0.023, 0.026]
Tau-368/tau N-Mid	0.06 [0.053, 0.083]	0.041 [0.035, 0.071]	0.055 [0.040, 0.071]

Data are presented as median [quartile 1, quartile 3] unless otherwise specified

^a^Two patients had progressed to AD dementia at clinical follow-up

^b^Significantly lower, relative to prodromal AD (*p* < 0.01)

^c^Significantly lower, relative to baseline (*p* < 0.01)

According to the selection criteria for CSF-controls, the levels of P-tau_181p_ and T-tau were significantly higher in AD patients. A clear group separation was also seen when using novel tau fragments, with AUC values for these close to 1. There were no significant differences in AUC values between groups (Online Resources 2, 3 and 4).

### Relationship between baseline PET measures and CSF tau

**Fig. 1:**
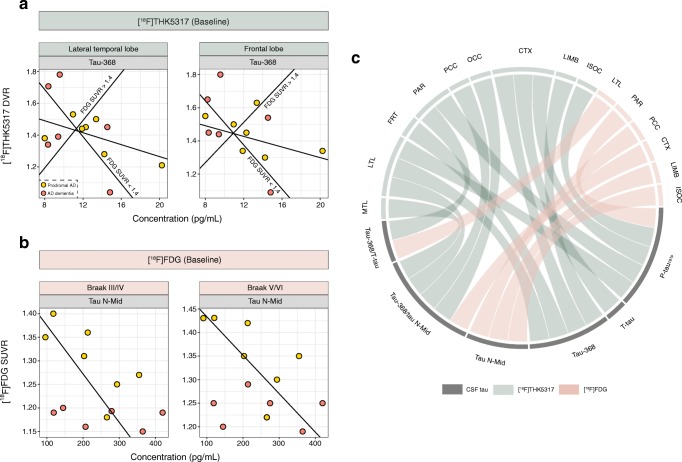
Relationship between [^18^F]THK5317 DVR and tau-368 levels (**a**) and between [^18^F]FDG SUVR and tau N-Mid levels (**b**) at baseline. FDG SUVR >1.4 and <1.4 indicate the multilinear regression fits for the significant interactions observed (i.e. a positive fit in those with an isocortical composite SUVR >1.4, and a negative fit in those with an isocortical composite SUVR <1.4). The chord diagram (**c**) shows the multilinear regression model findings, with each band indicating a significant relationship between CSF and PET measures. *MTL* medial temporal, *LTL* lateral temporal, *FRT* frontal, *PAR* parietal, *PCC* posterior cingulate, *OCC* occipital, *CTX* isocortical composite, *LIMB* Braak III/IV, *ISOC* Braak V/VI

Regression models for baseline [^18^F]THK5317 DVR (Fig. 1a, c; Online Resource 5) showed that tracer retention in the lateral temporal and parietal lobes, as well as in the isocortical composite and Braak V/VI ROIs, was associated with P-tau_181p_ levels, while T-tau levels were associated only with isocortical composite uptake. Tau-368 levels were associated with [^18^F]THK5317 DVR in the lateral temporal and frontal regions, as well as in the isocortical and Braak III/IV ROIs, and tau-368/tau N-Mid ratios were significantly associated with [^18^F]THK5317 DVR in the temporal ROIs, as well as in the parietal and occipital cortices. In contrast, tau-368/T-tau ratios were associated with [^18^F]THK5317 DVR only in the parietal lobe. Although the direction of these associations was negative, with the exception of tau ratios, as hypothesized, the relationship between CSF tau levels and [^18^F]THK5317 DVR varied according to the degree of glucose hypometabolism. Specifically, a significant interaction was observed between CSF tau measures and isocortical metabolism ([^18^F]FDG SUVR) whereby those with higher metabolism (SUVR >1.4; *n* = 7, five prodromal AD, two AD dementia) showed positive slopes for the above-described associations, while those with more impaired metabolism (SUVR <1.4; *n* = 6, four prodromal AD, two AD dementia) showed negative slopes, similar to the findings observed at the group level (in analyses using ratios, the additional AD dementia subject had an SUVR >1.4; Online Resource 6).

A significant association was observed between baseline parietal [^18^F]FDG SUVR and P-tau_181p_ levels (Fig. 1b, c; Online Resource 5). Significant associations were also found between tau N-Mid levels and [^18^F]FDG SUVR in the posterior cingulate, as well as in the Braak composite ROIs. Tau ratios were associated with lateral temporal (tau-368/T-tau) and isocortical composite (tau-368/tau N-Mid) metabolism. With respect to the strength of the associations, CSF tau on average explained 51.6% and 36.2% of the variance in regional [^18^F]THK5317 and [^18^F]FDG uptake values, respectively (average *R*^2^ 0.516 for [^18^F]THK5317, 0.362 for [^18^F]FDG; Online Resource 7). Tau-368 levels showed a stronger association than other CSF tau measures with regional [^18^F]THK5317 DVR (average *R*^2^ 0.443 and 0.651, respectively; Online Resource 7).

### Relationship between rate of change in PET measures and CSF tau

**Fig. 2:**
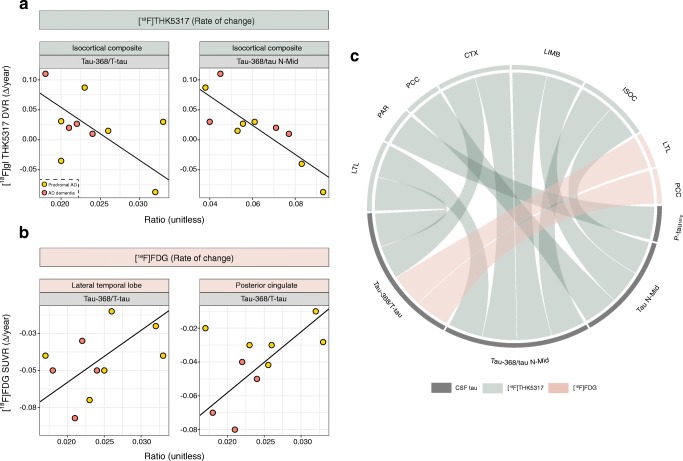
Relationships between the annual rate of change in [^18^F]THK5317 DVR and tau-368/T-tau ratio and tau N-Mid level (**a**) and between the annual rate of change in [^18^F]FDG SUVR and tau-368/T-tau ratio (**b**). The chord diagram (**c**) shows the multilinear regression model findings, with each band indicating a significant association. *MTL* medial temporal, *LTL* lateral temporal, *FRT* frontal, *PAR* parietal, *PCC* posterior cingulate, *OCC* occipital, *CTX* isocortical composite, *LIMB* Braak III/IV, *ISOC* Braak V/VI

Given the smaller number of participants who completed follow-up PET studies, the interaction term (isocortical [^18^F]FDG) used in baseline [^18^F]THK5317 models was omitted to avoid overfitting, with rate of change values simply regressed onto CSF tau values, adjusting for covariates. For [^18^F]THK5317 uptake, P-tau_181p_ levels were found to be associated with parietal rate of change, while tau N-Mid levels were associated with rates of change in parietal and Braak regions (Fig. 2a, c; Online Resource 8). Tau ratios were associated with the rate of change in [^18^F]THK5317 uptake in lateral temporal and isocortical composite regions, and tau-368/tau N-Mid ratios were also associated with the rate of change in Braak regions. The rates of change in [^18^F]FDG SUVR in lateral temporal and posterior cingulate regions were significantly associated with tau-368/T-tau ratios (Fig. 2b, c; Online Resource 8). Similar to the cross-sectional findings, CSF tau measures showed a stronger association with [^18^F]THK5317 uptake than with [^18^F]FDG uptake (average *R*^2^ 0.501 and 0.395, respectively; Online Resource 9).

### Concordance between tau biomarkers

**Fig. 3:**
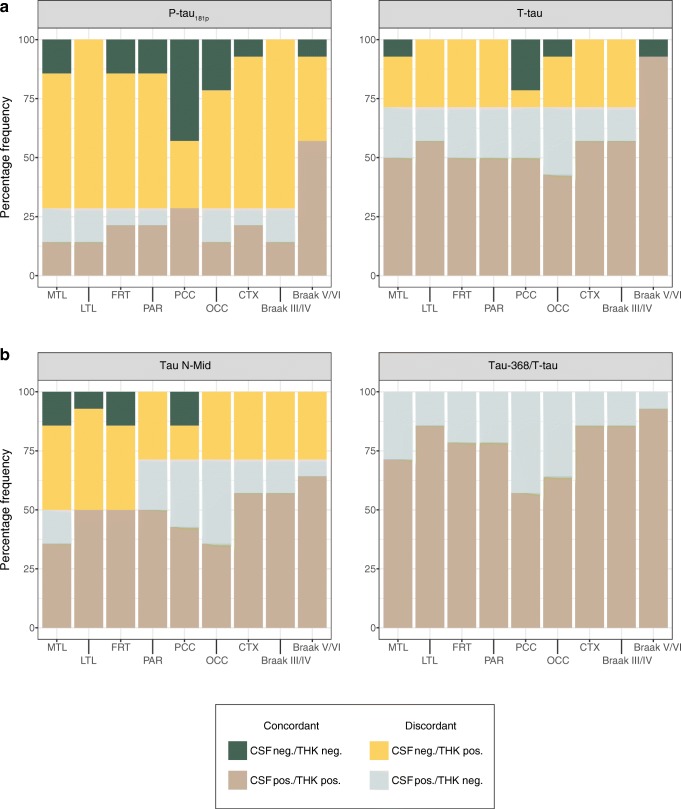
Concordance between binarized [^18^F]THK5317 uptake and CSF tau levels: INNOTEST tau (**a**), tau N-Mid and tau-368/T-tau (**b**). *MTL* medial temporal, *LTL* lateral temporal, *FRT* frontal, *PAR* parietal, *PCC* posterior cingulate, *OCC* occipital, *CTX* isocortical composite, *LIMB* Braak III/IV, *ISOC* Braak V/VI

Regional [^18^F]THK5317 DVR cut-off values were as follows: 1.11 (medial temporal), 1.28 (lateral temporal), 1.38 (parietal), 1.29 (posterior cingulate), 1.44 (frontal), 1.34 (isocortical composite), 1.22 (Braak III/IV), and 1.33 (Braak V/VI). Concordance findings are summarized in Fig. 3 and Online Resource 10. In all the AD patients (pooled), the average concordance between [^18^F]THK5317 uptake and INNOTEST tau measures was approximately 50%. Although a similar level of concordance was reached when using tau N-Mid, agreement was substantially higher when using the tau-368/T-tau ratio (79%). When using INNOTEST measures and tau N-Mid, discordance was primarily in the form of isolated [^18^F]THK5317 PET positivity. Only isolated CSF positivity was found when using tau-368/T-tau. This pattern held when subgroups were examined separately, although concordance was higher overall in AD dementia patients than in those with prodromal AD (Online Resources 11 and 12).

### Illustrative cases

The following four cases illustrate differing biomarker profiles. For CSF, concordance with [^18^F]THK5317 uptake was based on established tau measures (P-tau_181p_ and T-tau). Two patients showed concordant positive profiles while two showed isolated [^18^F]THK5317 positivity. When considering all ROIs, no patients showed isolated CSF positivity. These cases are presented to highlight various CSF tau profiles in the context of high cortical tau burden.

#### Prodromal AD

**Fig. 4:**
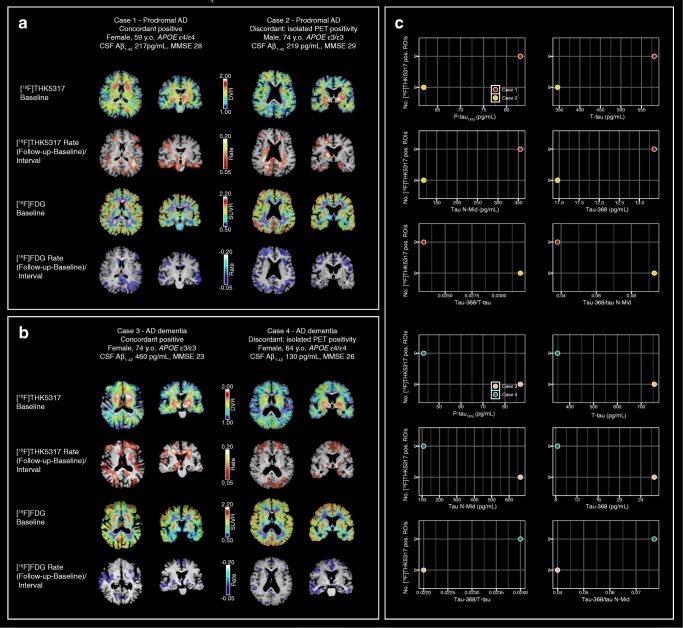
Imaging and CSF profiles in patients with a baseline diagnosis of prodromal AD (**a**, **c**), and AD dementia (**b**, **c**). Given the widespread use of P-tau_181p_ and T-tau in the clinical work-up of patients with dementia disorder, these measures were selected to define concordance (**a**, **b**). [^18^F]THK5317 positivity was considered present when a high percentage of ROIs showed DVR values above the defined cut-off values for THK-controls

The PET and CSF biomarker findings in two patients with prodromal AD are presented in Fig. 4a, c. Both patients showed high cortical [^18^F]THK5317 uptake (above cut-off values in nine ROIs (case 1) and eight of nine ROIs (case 2) and temporoparietal hypometabolism at baseline. Using P-tau_181p_ and T-tau, one patient (case 1) was concordant positive with the [^18^F]THK5317 findings, while the other (case 2) showed CSF tau concentrations below the cut-off values. One patient (case 1) showed increased [^18^F]THK5317 uptake, mainly in the temporal and parietal regions, with decreased [^18^F]FDG uptake in the lateral temporal cortex and the parietal cortex/posterior cingulate. The other patient (case 2) also showed increased [^18^F]THK5317 DVR in the medial parietal region, but with more widespread changes in metabolism, including in frontal areas. CSF tau N-Mid levels in the case 1 patient were nearly double those in the case 2 patient; tau ratios also showed a similar difference. Tau-368 levels, however, were similar. At clinical follow-up, the case 1 patient showed cognitive decline (in terms of MMSE score) and was diagnosed with AD dementia. In contrast, the case 2 patient showed relative cognitive stability and the diagnosis of prodromal AD was unchanged.

#### AD dementia

The PET and CSF biomarker findings in two patients with AD dementia are presented in Fig. 4b, c. In contrast to the first patient (case 3), who showed concordant positive biomarkers using INNOTEST tau, the second patient (case 4) showed a discordant profile, with isolated [^18^F]THK5317 positivity. At baseline, both patients showed high uptake of [^18^F]THK5317 in cortical regions (above regional cut-off values, except for a borderline DVR value in the posterior cingulate in the case 4 patient), and low [^18^F]FDG uptake in temporal, parietal and frontal regions. [^18^F]THK5317 uptake was higher at follow-up than at baseline in the frontal and parietal regions in both patients, with additional involvement of the temporal cortex in the case 3 patient. Both patients showed metabolic decline, predominantly in temporal areas. Large differences were seen in tau N-Mid and tau-368 levels and the tau-368/tau N-Mid ratio; however, both patients showed similar tau-368/T-tau ratios. No cognitive decline was seen in the case 3 patient at follow-up, while a decline in MMSE score of 2 points was seen in the case 4 patient.

## Discussion

In this retrospective longitudinal study, the relationship between PET and CSF tau biomarkers was found to vary in relation to the degree of isocortical glucose metabolism. While a negative association was observed in those with more globally decreased [^18^F]FDG uptake, positive slopes, in line with the findings of previous studies, were found in those with more preserved glucose metabolism. This interaction with [^18^F]FDG uptake was observed for both established and novel CSF tau fragments. The levels of all forms of CSF tau investigated were found to be negatively associated with [^18^F]FDG SUVR.

Given the unclear relationship between established CSF tau biomarkers and NFT burden [32, 33], increases in P-tau_181p_ and T-tau levels may in fact reflect increased tau secretion resulting from the spread of AD-affected cells at risk of tangle pathology [34], with increases in tau-368 levels reflecting an expansion in the pool of C-terminal fragments, possibly due to increased cleavage activity. In this way, higher levels of these biomarkers would precede the appearance of NFT pathology and neurodegenerative changes on [^18^F]FDG PET. This scenario may explain the positive associations with [^18^F]THK5317 uptake at baseline observed in this study and in previous studies using [^18^F]flortaucipir [4–7]. The inverse association between tau biomarker levels seen in patients with more impaired metabolism, by contrast, may reflect a decrease in the release of neuronal tau to the CSF. Indeed, several studies, including recent work using stable isotope labelling kinetics (SILK) to monitor the half-life and turnover rate of tau [35], have shown that, following an initial increase, CSF tau levels decrease in patients with symptomatic AD [15, 16, 36–38]. This may reflect a deceleration in neurodegenerative processes and/or a reduction in the number of viable neurons releasing tau. The inverse association with tau-368 levels may also reflect a slowing in the production of tau. Another intriguing hypothesis, however, is that this truncated tau form may be sequestered in NFTs and thus represent a more direct tangle marker than, for example, P-tau_181p_. Possibly, ratios incorporating tau-368 (particularly the tau-368/N-Mid ratio) might better reflect this process, in much the same that way Aβ_1–42_/Aβ_1–40_ better reflects Aβ pathology. The strong associations between tau-368 levels and baseline [^18^F]THK5317 uptake, and between tau-368 ratios and the change in [^18^F]THK5317 uptake, lend support to this hypothesis.

Previous studies addressing the relationship between cross-sectional [^18^F]FDG uptake and CSF tau levels (P-tau_181p_ and T-tau) in patients with AD have shown mixed results: although all have shown an inverse association, some have shown no significant association [39], significant associations only for P-tau_181p_ [40] and T-tau [41] levels, or significant associations for both [42]. In our cohort, a significant negative association was found only between P-tau_181p_ levels and parietal metabolism at baseline. Interestingly, P-tau_181p_ levels were also associated with the rate of change in [^18^F]THK5317 DVR in the parietal lobe, with this pattern (i.e. CSF tau levels associated with baseline metabolism and the rate of change in [^18^F]THK5317 uptake in the same ROI) also observed for the tau-368/T-tau ratio (lateral temporal), tau-368/tau N-Mid ratio (isocortical composite) and, most strikingly, tau N-Mid levels (posterior cingulate and Braak composite regions). Together, these findings suggest a nonlinear relationship between the accumulation of AD-type PHF tau and neurodegeneration [18, 43], similar to that observed for Aβ [44]. Further, our results suggest that tau N-Mid might prove suitable for capturing increases in tangle pathology and synaptic impairment, acting as both a state marker (reflecting the intensity of the disease process) and a stage marker (reflecting the cumulative extent of the degenerative process) [45].

The level of agreement between dichotomized [^18^F]THK5317 uptake and CSF tau levels was somewhat lower, overall, than that found in the only other study so far to have examined concordance between tau biomarkers ([^18^F]flortaucipir, and P-tau_181p_ and T-tau) [6]. This may relate to cohort-specific differences (age and CSF tau levels, as well as MMSE scores were higher and lower, respectively, in the study by Mattsson et al. [6], which also had a shorter time between CSF sampling and PET), ROI definition, tracer characteristics, and, possibly, the control subjects used to define tau PET cut-off levels. Similar to the study by Mattsson et al. [6], however, we also found higher concordance rates among AD dementia subjects than in subjects with prodromal AD. However, using single biomarkers concordance was markedly improved when using tau-368/T-tau. Although the findings for tau-368/T-tau and baseline [^18^F]THK5317 DVR were limited, this improved concordance, combined with the strong association between this measure and longitudinal increases in [^18^F]THK5317 retention, suggests that the tau-368/T-tau ratio may better reflect the shift between soluble and insoluble pools of tau than the single analytes (P-tau_181p_, T-tau, tau N-Mid) investigated. This would also probably hold true for tau-368 alone and as a ratio with tau N-Mid, although we were unable to directly test this in this study due the lack of a priori cut-off values or controls with both CSF and [^18^F]THK5317 PET data available.

With regard to the patterns of discordance, isolated [^18^F]THK5317 positivity was the predominant finding for P-tau_181p_, T-tau and tau N-Mid. By contrast, only isolated CSF positivity was found using tau-368/T-tau. Possibly, the isolated [^18^F]THK5317 positivity may reflect a deceleration in neurodegeneration and/or the spread of NFT pathology, as previously described [16, 37]. The pattern of discordance seen with tau-368/T-tau lends partial support to this idea, in that the spread of tangle pathology would be accompanied by a decrease in soluble C-terminal fragments, a process that would precede suprathreshold [^18^F]THK5317 DVR values. With regard to the cases presented, however, all four of the patients showed increases in [^18^F]THK5317 DVR and decreases in [^18^F]FDG SUVR, despite only the patients of cases 2 and 4 showing values below the thresholds for P-tau_181p_, T-tau and tau N-Mid. However, both these patients showed low baseline isocortical glucose metabolism. These observations, combined with our regression findings, suggest that tau biomarkers may initially follow an offset parallel upward course, followed by divergence, as CSF tau levels decline. Although this may indeed reflect the sequestration of tau fragments in NFTs, given the strong coupling between tangle pathology and neurodegeneration in AD [46], this decrease in soluble tau may relate less to a slowing of degenerative processes and more to a shift in the way tau is released from tangle-bearing cells [35].

Certain methodological limitations complicate the interpretation of the present findings. Firstly, in addition to the modest sample size, which precluded comparisons between subgroups (prodromal AD, AD dementia), not all participants completed follow-up PET. Also, due to the limited number of subjects, and the exploratory nature of this study, we did not correct for multiple comparisons. Moreover, the AD dementia subgroup represented a limited cross section of disease severity; with the exception of one AD dementia patient who had an MMSE score of 17, all had MMSE scores that placed them in the mildly impaired range. Ideally, Aβ-positive cognitively unimpaired elderly would also have been included so as to cover the entire AD continuum. In addition, although recent evidence indicates off-target binding of [^18^F]THK5351 to monoamine oxidase B (MAO-B) [47–49], it remains unclear how well these findings extrapolate to [^18^F]THK5317, given results showing that despite a similar cortical laminar distribution, the MAO-B-specific tracer [^3^H]deprenyl and [^3^H]THK5117 do not compete at the concentration ranges used in PET studies [50]. This, combined with the low levels of MAO-B in cortical regions [51], suggests that the DVR values reported here largely reflect specific binding of [^18^F]THK5317 to PHF-tau. Further, longitudinal CSF sampling performed concurrently with PET studies would have allowed a better understanding of the trajectories of tau biomarkers, in particular whether a decline in CSF tau levels precedes increases in [^18^F]THK5317 retention. Lastly, partial volume correction of longitudinal data would ideally have been performed using MRI at follow-up. However, given the low rates of atrophy reported within isocortical regions over comparable time intervals [52], this would not be expected to have had an important effect on our results.

### Conclusion

Although our findings are preliminary and subject to the above-mentioned caveats, they suggest that both positive and negative associations may exist between CSF and PET tau biomarkers, and that the directionality of their relationship may depend on the type of tau form measured, and/or an as-yet-unclear interplay between tau pathology and neurodegeneration. By comparison with P-tau_181p_ and T-tau, the novel fragments investigated in this study showed greater sensitivity toward findings with [^18^F]THK5317 and [^18^F]FDG PET, and improved concordance with binarized [^18^F]THK5317 measurements. CSF and PET tau biomarkers, however, probably capture different aspects of tau pathology, similar to that proposed for CSF Aβ_1–42_ and amyloid PET [53]. Further studies incorporating longitudinal CSF analysis and tau PET imaging in larger cohorts are required to clarify the findings reported here and to address their potential implications in the context of the recently proposed dichotomous classification scheme that seeks to combine tau biomarkers with those for Aβ and neurodegeneration [54].

## Electronic supplementary material

Online Resource 1 (DOC 60 kb)

Online Resource 2 (DOC 469 kb)

Online Resource 3 (DOC 34 kb)

Online Resource 4 (DOC 32 kb)

Online Resource 5 (DOC 46 kb)

Online Resource 6 (DOC 220 kb)

Online Resource 7 (DOC 48 kb)

Online Resource 8 (DOC 46 kb)

Online Resource 9 (DOC 46 kb)

Online Resource 10 (DOC 37 kb)

Online Resource 11 (DOC 37 kb)

Online Resource 12 (DOC 37 kb)

## References

[1] Blennow K, Hampel H, Weiner M, Zetterberg H (2010). Cerebrospinal fluid and plasma biomarkers in Alzheimer disease. Nat Rev Neurol.

[2] Chien DT, Bahri S, Szardenings AK, Walsh JC, Mu F, Su MY (2013). Early clinical PET imaging results with the novel PHF-tau radioligand [F-18]-T807. J Alzheimers Dis.

[3] Xia CF, Arteaga J, Chen G, Gangadharmath U, Gomez LF, Kasi D (2013). [(18)F]T807, a novel tau positron emission tomography imaging agent for Alzheimer's disease. Alzheimers Dement.

[4] Chhatwal JP, Schultz AP, Marshall GA, Boot B, Gomez-Isla T, Dumurgier J (2016). Temporal T807 binding correlates with CSF tau and phospho-tau in normal elderly. Neurology.

[5] Gordon BA, Friedrichsen K, Brier M, Blazey T, Su Y, Christensen J (2016). The relationship between cerebrospinal fluid markers of Alzheimer pathology and positron emission tomography tau imaging. Brain.

[6] Mattsson N, Scholl M, Strandberg O, Smith R, Palmqvist S, Insel PS (2017). 18F-AV-1451 and CSF T-tau and P-tau as biomarkers in Alzheimer's disease. EMBO Mol Med.

[7] La Joie R, Bejanin A, Fagan AM, Ayakta N, Baker SL, Bourakova V (2018). Associations between [(18)F]AV1451 tau PET and CSF measures of tau pathology in a clinical sample. Neurology.

[8] Blennow K, Wallin A, Agren H, Spenger C, Siegfried J, Vanmechelen E (1995). Tau protein in cerebrospinal fluid: a biochemical marker for axonal degeneration in Alzheimer disease?. Mol Chem Neuropathol.

[9] Olsson A, Vanderstichele H, Andreasen N, De Meyer G, Wallin A, Holmberg B (2005). Simultaneous measurement of beta-amyloid(1-42), total tau, and phosphorylated tau (Thr181) in cerebrospinal fluid by the xMAP technology. Clin Chem.

[10] Meredith JE, Sankaranarayanan S, Guss V, Lanzetti AJ, Berisha F, Neely RJ (2013). Characterization of novel CSF tau and ptau biomarkers for Alzheimer's disease. PLoS One.

[11] Saint-Aubert L, Almkvist O, Chiotis K, Almeida R, Wall A, Nordberg A (2016). Regional tau deposition measured by [(18)F]THK5317 positron emission tomography is associated to cognition via glucose metabolism in Alzheimer's disease. Alzheimers Res Ther.

[12] Jonasson M, Wall A, Chiotis K, Saint-Aubert L, Wilking H, Sprycha M (2016). Tracer kinetic analysis of (S)-18F-THK5117 as a PET tracer for assessing tau pathology. J Nucl Med.

[13] Lemoine L, Saint-Aubert L, Marutle A, Antoni G, Eriksson JP, Ghetti B (2015). Visualization of regional tau deposits using (3)H-THK5117 in Alzheimer brain tissue. Acta Neuropathol Commun.

[14] Harada R, Okamura N, Furumoto S, Furukawa K, Ishiki A, Tomita N (2015). [(18)F]THK-5117 PET for assessing neurofibrillary pathology in Alzheimer's disease. Eur J Nucl Med Mol Imaging.

[15] Toledo JB, Xie SX, Trojanowski JQ, Shaw LM (2013). Longitudinal change in CSF tau and Abeta biomarkers for up to 48 months in ADNI. Acta Neuropathol.

[16] Fagan AM, Xiong C, Jasielec MS, Bateman RJ, Goate AM, Benzinger TL (2014). Longitudinal change in CSF biomarkers in autosomal-dominant Alzheimer's disease. Sci Transl Med.

[17] Chiotis K, Saint-Aubert L, Savitcheva I, Jelic V, Andersen P, Jonasson M (2016). Imaging in-vivo tau pathology in Alzheimer's disease with THK5317 PET in a multimodal paradigm. Eur J Nucl Med Mol Imaging.

[18] Chiotis K, Saint-Aubert L, Rodriguez-Vieitez E, Leuzy A, Almkvist O, Savitcheva I (2018). Longitudinal changes of tau PET imaging in relation to hypometabolism in prodromal and Alzheimer's disease dementia. Mol Psychiatry.

[19] McKhann GM, Knopman DS, Chertkow H, Hyman BT, Jack CR, Kawas CH (2011). The diagnosis of dementia due to Alzheimer's disease: recommendations from the National Institute on Aging-Alzheimer's Association workgroups on diagnostic guidelines for Alzheimer's disease. Alzheimers Dement.

[20] Petersen RC, Smith GE, Waring SC, Ivnik RJ, Tangalos EG, Kokmen E (1999). Mild cognitive impairment: clinical characterization and outcome. Arch Neurol.

[21] Dubois B, Feldman HH, Jacova C, Hampel H, Molinuevo JL, Blennow K (2014). Advancing research diagnostic criteria for Alzheimer's disease: the IWG-2 criteria. Lancet Neurol.

[22] Zhang Z, Song M, Liu X, Kang SS, Kwon IS, Duong DM (2014). Cleavage of tau by asparagine endopeptidase mediates the neurofibrillary pathology in Alzheimer's disease. Nat Med.

[23] Muller-Gartner HW, Links JM, Prince JL, Bryan RN, McVeigh E, Leal JP (1992). Measurement of radiotracer concentration in brain gray matter using positron emission tomography: MRI-based correction for partial volume effects. J Cereb Blood Flow Metab.

[24] Logan J, Fowler JS, Volkow ND, Wang GJ, Ding YS, Alexoff DL (1996). Distribution volume ratios without blood sampling from graphical analysis of PET data. J Cereb Blood Flow Metab.

[25] Hammers A, Allom R, Koepp MJ, Free SL, Myers R, Lemieux L (2003). Three-dimensional maximum probability atlas of the human brain, with particular reference to the temporal lobe. Hum Brain Mapp.

[26] Braak H, Braak E (1991). Neuropathological stageing of Alzheimer-related changes. Acta Neuropathol.

[27] Leuzy A, Chiotis K, Hasselbalch SG, Rinne JO, de Mendonca A, Otto M (2016). Pittsburgh compound B imaging and cerebrospinal fluid amyloid-β in a multicentre European memory clinic study. Brain.

[28] Clark CM, Schneider JA, Bedell BJ, Beach TG, Bilker WB, Mintun MA (2011). Use of florbetapir-PET for imaging beta-amyloid pathology. JAMA.

[29] Kloke JD, Mckean JW (2012). Rfit: rank-based estimation for linear models. R J.

[30] Preacher KJ, Curran PJ, Bauer DJ (2006). Computational tools for probing interactions in multiple linear regression, multilevel modeling, and latent curve analysis. J Educ Behav Stat.

[31] Robin X, Turck N, Hainard A, Tiberti N, Lisacek F, Sanchez JC (2011). pROC: an open-source package for R and S+ to analyze and compare ROC curves. BMC Bioinformatics.

[32] Engelborghs S, Sleegers K, Cras P, Brouwers N, Serneels S, De Leenheir E (2007). No association of CSF biomarkers with APOEepsilon4, plaque and tangle burden in definite Alzheimer's disease. Brain.

[33] Buerger K, Alafuzoff I, Ewers M, Pirttila T, Zinkowski R, Hampel H (2007). No correlation between CSF tau protein phosphorylated at threonine 181 with neocortical neurofibrillary pathology in Alzheimer's disease. Brain.

[34] Zetterberg H (2018). Tauomics and kinetics in human neurons and biological fluids. Neuron.

[35] Sato C, Barthelemy NR, Mawuenyega KG, Patterson BW, Gordon BA, Jockel-Balsarotti J (2018). Tau kinetics in neurons and the human central nervous system. Neuron.

[36] Seppala TT, Koivisto AM, Hartikainen P, Helisalmi S, Soininen H, Herukka SK (2011). Longitudinal changes of CSF biomarkers in Alzheimer's disease. J Alzheimers Dis.

[37] McDade E, Wang G, Gordon BA, Hassenstab J, Benzinger TLS, Buckles V, et al. Longitudinal cognitive and biomarker changes in dominantly inherited Alzheimer disease. Neurology. 2018;91:e1295–306. 10.1212/WNL.0000000000006277. 10.1212/WNL.0000000000006277 PMC6177272 30217935

[38] Sutphen CL, McCue L, Herries EM, Xiong C, Ladenson JH, Holtzman DM (2018). Longitudinal decreases in multiple cerebrospinal fluid biomarkers of neuronal injury in symptomatic late onset Alzheimer's disease. Alzheimers Dement.

[39] Jagust WJ, Landau SM, Shaw LM, Trojanowski JQ, Koeppe RA, Reiman EM (2009). Relationships between biomarkers in aging and dementia. Neurology.

[40] Fellgiebel A, Siessmeier T, Scheurich A, Winterer G, Bartenstein P, Schmidt LG (2004). Association of elevated phospho-tau levels with Alzheimer-typical 18F-fluoro-2-deoxy-D-glucose positron emission tomography findings in patients with mild cognitive impairment. Biol Psychiatry.

[41] Haense C, Buerger K, Kalbe E, Drzezga A, Teipel SJ, Markiewicz P (2008). CSF total and phosphorylated tau protein, regional glucose metabolism and dementia severity in Alzheimer's disease. Eur J Neurol.

[42] Ceravolo R, Borghetti D, Kiferle L, Tognoni G, Giorgetti A, Neglia D (2008). CSF phosporylated TAU protein levels correlate with cerebral glucose metabolism assessed with PET in Alzheimer's disease. Brain Res Bull.

[43] Leuzy A, Rodriguez-Vieitez E, Saint-Aubert L, Chiotis K, Almkvist O, Savitcheva I (2018). Longitudinal uncoupling of cerebral perfusion, glucose metabolism, and tau deposition in Alzheimer's disease. Alzheimers Dement.

[44] Forster S, Grimmer T, Miederer I, Henriksen G, Yousefi BH, Graner P (2012). Regional expansion of hypometabolism in Alzheimer's disease follows amyloid deposition with temporal delay. Biol Psychiatry.

[45] Blennow K, Hampel H (2003). CSF markers for incipient Alzheimer's disease. Lancet Neurol.

[46] Spillantini MG, Goedert M (2013). Tau pathology and neurodegeneration. Lancet Neurol.

[47] Ng KP, Pascoal TA, Mathotaarachchi S, Therriault J, Kang MS, Shin M (2017). Monoamine oxidase B inhibitor, selegiline, reduces 18F-THK5351 uptake in the human brain. Alzheimers Res Ther.

[48] Harada R, Ishiki A, Kai H, Sato N, Furukawa K, Furumoto S (2018). Correlations of 18F-THK5351 PET with post-mortem burden of tau and astrogliosis in Alzheimer's disease. J Nucl Med.

[49] Lemoine L, Gillberg PG, Svedberg M, Stepanov V, Jia Z, Huang J (2017). Comparative binding properties of the tau PET tracers THK5117, THK5351, PBB3, and T807 in postmortem Alzheimer brains. Alzheimers Res Ther.

[50] Lemoine L, Saint-Aubert L, Nennesmo I, Gillberg PG, Nordberg A (2017). Cortical laminar tau deposits and activated astrocytes in Alzheimer's disease visualised by (3)H-THK5117 and (3)H-deprenyl autoradiography. Sci Rep.

[51] Tong J, Meyer JH, Furukawa Y, Boileau I, Chang LJ, Wilson AA (2013). Distribution of monoamine oxidase proteins in human brain: implications for brain imaging studies. J Cereb Blood Flow Metab.

[52] Henneman WJ, Sluimer JD, Barnes J, van der Flier WM, Sluimer IC, Fox NC (2009). Hippocampal atrophy rates in Alzheimer disease: added value over whole brain volume measures. Neurology.

[53] Mattsson N, Insel PS, Donohue M, Landau S, Jagust WJ, Shaw LM (2015). Independent information from cerebrospinal fluid amyloid-beta and florbetapir imaging in Alzheimer's disease. Brain.

[54] Jack CR, Bennett DA, Blennow K, Carrillo MC, Feldman HH, Frisoni GB (2016). A/T/N: an unbiased descriptive classification scheme for Alzheimer disease biomarkers. Neurology.

