# Supplementary material for: Longitudinal tau and metabolic PET imaging in relation to novel CSF tau measures in Alzheimer’s disease
Source: Eur J Nucl Med Mol Imaging. 2019 Jan 4;46(5):1152–63. doi: 10.1007/s00259-018-4242-6 (PMC6451715; doi:10.1007/s00259-018-4242-6)
Supplement: Supplementary file 1 — (DOC 60 kb) [file 259_2018_4242_MOESM1_ESM.doc]

**CSF samples**

CSF samples used in the present study had been obtained as part of the routine clinical work up of dementia disorder patients seen at Karolinska University Hospital, Huddinge, Sweden. These samples had been obtained by lumbar puncture, with a total of 10 mL drawn and stored in polypropylene tubes. After discarding the first 0.5 mL, samples were centrifuged at 1500 × g (3000 – 4000 rpm) for 10 min (+4 °C) and stored at −80 °C in 1 ml portions pending biochemical analysis, without being thawed or refrozen. CSF aliquots remaining following the original analyses described above were stored on dry ice and sent via express courier to the Clinical Neurochemistry Laboratory, University of Gothenburg, Mölndal, Sweden. CSF tau was quantified using three approaches: using the commercially available Fujirebio INNOTEST kit (mid-domain), an ELISA based method targeting N-terminus and mid-domain (tau N-Mid), and a single molecule array based method targeting C-terminus and mid-domain regions (tau-368). Levels of Aβ_1-42_ were also determined.

**INNOTEST P-tau_181p_ , T-tau, and MSD Aβ_1-42_**

Tau (P-tau_181p_, T-tau) and Aβ_1-42_ were determined using INNOTEST ELISAs and MSD electrochemiluminescence analyses, respectively, according to the manufacturer’s protocols.

**ELISA tau N-Mid**

The ELISA based Tau N-Mid assay was performed as previously described (manuscript in preparation). In brief, Tau12 (Nordic Biosite, binding region aa9-18) was used as coating antibody and, as primary, a combination of biotinylated HT7 (Thermo Scientific, aa159-163) and BT2 (Thermo Scientific, aa194-198). For detection, enhanced streptavidin-HRP complex was used. Full-length recombinant Tau 441 2N4R (rPeptide) was used as calibrator.

**ELISA tau N-Mid assay validation**

***Method***

For assay validation, several plates were run at different occasions. Calibrators and low and high quality control (QC) samples were included in duplicate. The lower and upper limits of quantification (LLOQ and ULOQ, respectively) were determined by analyzing the deviation from the true value of each calibrator point. Limit of detection (LOD)s was determined by analyzing 16 duplicates of the blank and by adding 3 standard deviations to the mean blank signal (16 replicates/one plate). Precision is defined by the calculated standard deviation (SD_r_) and the variation coefficient (CV_r_) using one-way ANOVA in accordance with ISO 5257-2.

***Results***

In the N-mid assay, LLOQ and ULOQ were respectively 33 pg/mL and 6250 pg/mL, determined as described above. The CV% for the back calculated concentrations of the data from the calibrator curve was < 20% for the 5 points in the central part of the standard curve, where the samples concentrations are distributed. Within- and between-plate variability were measured over 6 runs and were, respectively, 9.2% and 10.7% for the high QC sample and 13.6% and 21.4% for the low QC sample. LOD was 7 pg/mL. Recovery after step-wise dilutions (1:2, 1:4) was between 94.8% and 87.6%.

**SIMOA tau-368**

The method has been described previously (manuscript in preparation). Tau 368 was measured through single-molecule array on a Simoa HD-1 analyzer (Simoa, Quanterix, Lexington, MA, USA). Magnetic beads (Quanterix, Lexington, MA, USA) were conjugated with capture antibody anti-Tau368[2] according to supplier’s conjugation protocol. Tau 1-368 recombinant protein [2] was series diluted and used as calibrator. As detection antibody, biotin-labeled KJ9A (Sigma) was used.

**SIMOA tau-368 assay validation**

**Method**

All samples from each individual patient were measured within the same run. LLOQ and ULOQ were determined by analyzing the deviation from the true value of each calibration point. The CV for the back-calculated concentrations of the data from the calibrator curve has to be <25% at LLOQ and ULOQ, while <20% in between. The Limit of Detection (LOD) was determined by analyzing 16 duplicates of the blank and by adding 3 standard deviations to the mean blank signal (16 replicates/one plate) the concentration is calculated using the calibration curve. Precision is defined by the calculated standard deviation (SD_r_) and the variation coefficient (CV_r_) using One-way ANOVA in accordance with ISO 5257-2.

**Results**

The calibration range for the assay was set to 4.88-625 pg/mL. The LOD was determined by analyzing 16 duplicates of the blank. The mean AEB value was 0.0675 and the standard deviation was 0.01158. Adding three SD resulted in an AEB of 0.1023, which corresponds to a LOD of 3.6 pg/mL. Calibration curve data from five assay runs were used to determine the ULOQ and LLOQ where the relative error of the back-calculated concentrations for the calibrators was plotted as a function of concentration (data not shown). LLOQ was set to 4.88 pg/mL and ULOQ to 625 pg/mL. The precision (CV_r_) was < 7.9% and the between-run CV_Rw_ was below 12.3%.

**References**

[1] Portelius E, Zetterberg H, Skillback T, Tornqvist U, Andreasson U, Trojanowski JQ, et al. Cerebrospinal fluid neurogranin: relation to cognition and neurodegeneration in Alzheimer's disease. Brain. 2015;138:3373-85.

[2] Zhang Z, Song M, Liu X, Kang SS, Kwon IS, Duong DM, et al. Cleavage of tau by asparagine endopeptidase mediates the neurofibrillary pathology in Alzheimer's disease. Nat Med. 2014;20:1254-62.
