# Supplementary material for: Longitudinal tau and metabolic PET imaging in relation to novel CSF tau measures in Alzheimer’s disease
Source: Eur J Nucl Med Mol Imaging. 2019 Jan 4;46(5):1152–63. doi: 10.1007/s00259-018-4242-6 (PMC6451715; doi:10.1007/s00259-018-4242-6)
Supplement: Supplementary file 2 — (DOC 469 kb) [file 259_2018_4242_MOESM2_ESM.doc]

**Online Resource 2.** Scatterplots for CSF tau measures in AD patients and CSF-controls

**
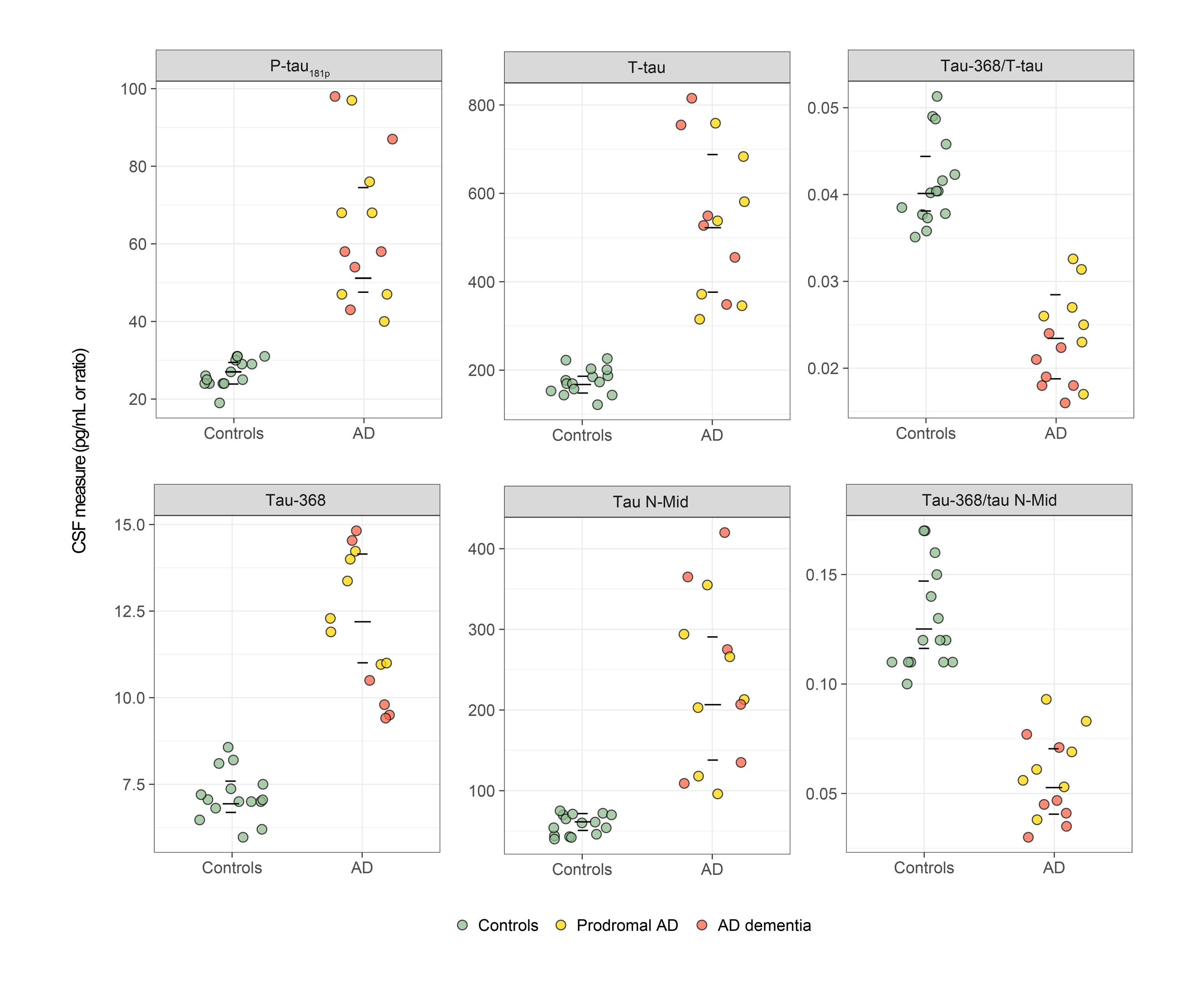
**CSF-controls were defined as such due normal basic and (cell count, albumin ratio, IgG index) and core (Aβ_1-42_/Aβ_1-40_, P-tau_181p_, and T-tau) CSF biomarkers.
