# Supplementary material for: Longitudinal tau and metabolic PET imaging in relation to novel CSF tau measures in Alzheimer’s disease
Source: Eur J Nucl Med Mol Imaging. 2019 Jan 4;46(5):1152–63. doi: 10.1007/s00259-018-4242-6 (PMC6451715; doi:10.1007/s00259-018-4242-6)
Supplement: Supplementary file 3 — (DOC 34 kb) [file 259_2018_4242_MOESM3_ESM.doc]

***Online Resource 3.*** *CSF tau findings for AD patients and CSF-controls*

|  | P-tau_181p_ | T-tau | Tau N-Mid | Tau-368 | Tau 368/T-tau | Tau 368/tau N-Mid |
| --- | --- | --- | --- | --- | --- | --- |
| AD | 58 [47, 76] | 529 [372, 683] | 213 [135, 294] | 12 [11, 14] | 0.023 [0.018, 0.026] | 0.056 [0.041, 0.071] |
| CSF-controls | 26 [24, 30] ^a^ | 173 [155, 194] ^a^ | 60 [45, 70] ^a^ | 7.1 [7, 7.4] ^a^ | 0.04 [0.038, 0.044] ^b^ | 0.190 [0.112, 0.143] ^b^ |

Data are presented as median [Quartile 1, Quartile 3]. ^a^ Significantly lower, relative to AD (*p* < 0.001); ^b^ Significantly higher, relative to AD (*p* < 0.001).
