# Supplementary material for: Longitudinal tau and metabolic PET imaging in relation to novel CSF tau measures in Alzheimer’s disease
Source: Eur J Nucl Med Mol Imaging. 2019 Jan 4;46(5):1152–63. doi: 10.1007/s00259-018-4242-6 (PMC6451715; doi:10.1007/s00259-018-4242-6)
Supplement: Supplementary file 4 — (DOC 32 kb) [file 259_2018_4242_MOESM4_ESM.doc]

**Online Resource 4.** ROC derived AUC values for CSF tau measures for the comparison AD vs CSF-controls

|  | P-tau_181p_ | T-tau | Tau N-Mid | Tau-368 | Tau 368/T-tau | Tau 368/tau N-Mid |
| --- | --- | --- | --- | --- | --- | --- |
| AUC | 0.972 [0.924 - 1] | 0.985 [0.950 - 1] | 0.964 [0.910 - 1] | 0.985 [0.950 - 1] | 0.944 [0.870 - 1] | 0.924 [0.864 - 1] |

Data are presented as AUC values [95% confidence interval], derived from comparisons between AD patients and CSF-controls. No significant differences were found between these values.
