# Supplementary material for: Longitudinal tau and metabolic PET imaging in relation to novel CSF tau measures in Alzheimer’s disease
Source: Eur J Nucl Med Mol Imaging. 2019 Jan 4;46(5):1152–63. doi: 10.1007/s00259-018-4242-6 (PMC6451715; doi:10.1007/s00259-018-4242-6)
Supplement: Supplementary file 6 — (DOC 220 kb) [file 259_2018_4242_MOESM6_ESM.doc]

**Online Resource 6.** Demographic, clinical, and CSF data by [^18^F]FDG sub-group

|  | [^18^F]FDG > 1.4 SUVR | [^18^F]FDG < 1.4 SUVR |
| --- | --- | --- |
| Prod-AD/AD dementia, no. | 4/3 | 3/4 |
| Age at baseline, years | 74 [62, 75] | 65 [59, 69] |
| Male, no. (%) | 2 (29%) | 2 (29%) |
| APOE ε4, no. (%) | 4 (57%) | 5 (71%) |
| MMSE at baseline | 28 [25, 29] | 23 [22, 27] |
| [^18^F]THK5317, no. + ROIs | 8 [4.5, 9] | 7 [6, 9] |
| Aβ_1-42_, pg/mL ­ | 217 [188, 336] | 236 [198, 345] |
| P-tau_181p_, pg/mL | 47 [45, 63] | 76 [63, 92] |
| T-tau, pg/mL | 372 [374, 555] | 683 [553, 757] |
| Tau N-Mid, pg/mL ­ | 203 [114, 281] | 275 [240, 330] |
| Tau-368, pg/mL ­ | 11 [9, 13] | 14 [11, 15] |
| Tau-368/T-tau | 0.024 [0.02, 0.029] | 0.019 [0.018,0.024] |
| Tau-368/tau N-Mid | 0.061 [0.043, 0.080] | 0.053 [0.038, 0.062] |

Data are presented as no., no. (%), or median [Quartile 1, Quartile 3].
