# Supplementary material for: Longitudinal tau and metabolic PET imaging in relation to novel CSF tau measures in Alzheimer’s disease
Source: Eur J Nucl Med Mol Imaging. 2019 Jan 4;46(5):1152–63. doi: 10.1007/s00259-018-4242-6 (PMC6451715; doi:10.1007/s00259-018-4242-6)
Supplement: Supplementary file 7 — (DOC 48 kb) [file 259_2018_4242_MOESM7_ESM.doc]

**Online Resource 7.** Coefficients of determination between CSF tau measures and PET measures at baseline

|  | MTL | | LTL | | FRT | | PCC | | PAR | | OCC | | CTX | | LIMB | | ISOC | |
| --- | --- | --- | --- | --- | --- | --- | --- | --- | --- | --- | --- | --- | --- | --- | --- | --- | --- | --- |
|  | THK | FDG | THK | FDG | THK | FDG | THK | FDG | THK | FDG | THK | FDG | THK | FDG | THK | FDG | THK | FDG |
| P-tau_181p_ |  |  | 0.472  * |  |  |  |  |  | 0.390  ** | 0.242  * |  |  | 0.445  ** |  |  |  | 0.466  ** |  |
| T-tau |  |  |  |  |  |  |  |  |  |  |  |  | 0.296  * |  |  |  |  |  |
| Tau N-Mid |  |  |  |  |  |  |  | 0.234  * |  |  |  |  |  |  |  | 0.358  * |  | 0.492  ** |
| Tau-368 |  |  | 0.684  ** |  | 0.660  ** |  |  |  |  |  |  |  | 0.735  ** |  | 0.524  ** |  |  |  |
| Tau 368/T-tau |  |  |  | 0.394  ** |  |  |  |  | 0.239  ** |  |  |  |  |  |  |  |  |  |
| Tau 368/tau N-Mid | 0.641  ** |  | 0.562  ** |  |  |  | 0.523  ** |  |  |  | 0.391  * |  |  | 0.448  * |  |  |  |  |

THK, [^18^F]THK5317 PET; FDG, [^18^F]FDG PET; *, *p* < 0.05; **, *p* < 0.01; Blank cells indicate statistically insignificant results. MTL, medial temporal lobe; LTL, lateral temporal lobe; FRT, frontal lobe; PAR, parietal lobe; PCC, posterior cingulate; OCC, occipital lobe; CTX, isocortical composite; LIMB, Braak III/IV; ISOC, Braak V/VI.
