# Supplementary material for: Longitudinal tau and metabolic PET imaging in relation to novel CSF tau measures in Alzheimer’s disease
Source: Eur J Nucl Med Mol Imaging. 2019 Jan 4;46(5):1152–63. doi: 10.1007/s00259-018-4242-6 (PMC6451715; doi:10.1007/s00259-018-4242-6)
Supplement: Supplementary file 10 — (DOC 37 kb) [file 259_2018_4242_MOESM10_ESM.doc]

**Online Resource 10.** Concordances between [^18^F]THK5317 and CSF tau measures in AD patients (prodromal and dementia pooled)

|  | MTL | LTL | FRT | PCC | PAR | OCC | CTX | LIMB | ISOC |
| --- | --- | --- | --- | --- | --- | --- | --- | --- | --- |
| P-tau_181p_ | 4/2 (43%)  2/6 (57%) | 4/0 (29%)  2/8 (71%) | 4/4 (57%)  2/4 (43%) | 4/6 (71%)  2/2 (29%) | 4/2 (43%)  3/5 (57%) | 4/5 (64%)  2/3 (36%) | 4/3 (50%)  2/5 (50%) | 4/2 (43%)  2/6 (57%) | 5/2 (50%)  1/6 (50%) |
| T-tau | 7/1 (57%)  3/3 (43%) | 8/0 (57%)  2/4 (43%) | 6/0 (43%)  4/4 (57%) | 5/3 (57%)  5/1 (43%) | 6/0 (43%)  4/4 (57%) | 6/1 (50%)  4/3 (50%) | 7/0 (50%)  4/3 (50%) | 8/0 (57%)  2/4 (43%) | 9/0 (64%)  1/4 (36%) |
| Tau N-Mid | 5/3 (57%)  1/5 (43%) | 6/1 (50%)  0/7 (50%) | 5/2 (50%)  1/6 (50%) | 5/4 (64%)  1/4 (36%) | 7/0 (50%)  4/3 (50%) | 5/3 (57%)  4/2 (43%) | 5/1 (43%)  1/7 (57%) | 6/2 (57%)  0/6 (43%) | 6/1 (50%)  0/7 (50%) |
| Tau 368/T-tau | 10/0 (71%)  4/0 (29%) | 13/0 (93%)  1/0 (7%) | 11/0 (71%)  3/0 (29%) | 10/0 (71%)  4/0 (29%) | 11/0 (79%)  3/0 (21%) | 9/0 (64%)  5/0 (36%) | 12/0 (86%)  2/0 (14%) | 12/0 (86%)  2/0 (14%) | 13/0 (93%)  1/0 (7%) |

The top and bottom rows within each cell indicate, respectively, the number and percentage of concordant (CSF+THK+/CSF-THK-) and discordant (CSF+THK-/CSF-THK+) subjects. MTL, medial temporal lobe; LTL, lateral temporal lobe; FRT, frontal lobe; PAR, parietal lobe; PCC, posterior cingulate; OCC, occipital lobe; CTX, isocortical composite; LIMB, Braak III/IV; ISOC, Braak V/VI.
