# Supplementary material for: Longitudinal tau and metabolic PET imaging in relation to novel CSF tau measures in Alzheimer’s disease
Source: Eur J Nucl Med Mol Imaging. 2019 Jan 4;46(5):1152–63. doi: 10.1007/s00259-018-4242-6 (PMC6451715; doi:10.1007/s00259-018-4242-6)
Supplement: Supplementary file 12 — (DOC 37 kb) [file 259_2018_4242_MOESM12_ESM.doc]

**Additional file 12. Concordances between [^18^F]THK5317 and CSF tau measures in AD dementia patients**

|  | MTL | LTL | FRT | PCC | PAR | OCC | CTX | LIMB | ISOC |
| --- | --- | --- | --- | --- | --- | --- | --- | --- | --- |
| P-tau_181p_ | 4/0 (57%)  2/1 (43%) | 5/0 (71%)  1/1 (29%) | 4/0 (57%)  2/1 (43%) | 4/1 (57%)  2/0 (43%) | 5/0 (71%)  1/1 (29%) | 4/0 (57%)  2/1 (43%) | 5/0 (71%)  1/1 (29%) | 5/0 (71%)  1/1 (29%) | 5/0 (71%)  1/1 (29%) |
| T-tau | 4/1 (71%)  1/1 (20%) | 4/0 (57%)  1/2 (43%) | 4/1 (71%)  1/1 (20%) | 4/2 (86%)  1/0 (14%) | 4/0 (57%)  1/2 (43%) | 4/1 (71%)  1/1 (20%) | 4/0 (57%)  1/2 (43%) | 4/0 (57%)  1/2 (43%) | 4/0 (57%)  1/2 (43%) |
| Tau N-Mid | 3/1 (57%)  1/2 (43%) | 4/0 (57%)  0/3 (43%) | 4/1 (71%)  0/2 (29%) | 4/2 (71%)  0/1 (29%) | 3/0 (43%)  1/3 (57%) | 3/1 (57%)  1/2 (43%) | 3/0 (57%)  1/3 (43%) | 3/0 (57%)  1/3 (43%) | 3/0 (57%)  1/3 (43%) |
| Tau 368/T-tau | 5/0 (71%)  2/0 (29%) | 6/0 (86%)  1/0 (14%) | 5/0 (71%)  2/0 (29%) | 4/0 (57%)  3/0 (43%) | 6/0 (86%)  1/0 (14%) | 6/0 (86%)  1/0 (14%) | 6/0 (71%)  1/0 (29%) | 6/0 (71%)  1/0 (29%) | 6/0 (71%)  1/0 (29%) |

The top and bottom rows within each cell indicate, respectively, the number and percentage of concordant (CSF+THK+/CSF-THK-) and discordant (CSF+THK- /CSF-THK+) subjects. MTL, medial temporal lobe; LTL, lateral temporal lobe; FRT, frontal lobe; PAR, parietal lobe; PCC, posterior cingulate; OCC, occipital lobe; CTX, isocortical composite; LIMB, Braak III/IV; ISOC, Braak V/VI.
